# Morbidity and mortality after general surgery in heart and lung transplant patients

**DOI:** 10.1016/j.sopen.2019.12.001

**Published:** 2020-01-11

**Authors:** Alek Zywot, Amber L. Turner, Joanna Sesti, Russell C. Langan, Andrew Nguyen, Andreas R. de Biasi, Siva Raja, Usman Ahmad, Subroto Paul

**Affiliations:** a Thoracic Surgical Services, RWJBarnabas Health, Saint Barnabas Medical Center, West Orange, NJ; b Department of Surgery, RWJBarnabas Health, Saint Barnabas Medical Center, Livingston, NJ; c Department of Surgery, Rutgers New Jersey Medical School, Newark, NJ; d Department of Surgical Oncology, Rutgers Cancer Institute of New Jersey, New Brunswick, NJ; e Department of Cardiothoracic Surgery, Stanford University, Stanford, CA; f Department of Thoracic and Cardiovascular Surgery, Heart and Vascular Institute, Cleveland Clinic, Cleveland, OH; g Transplantation Institute, Cleveland Clinic, Cleveland, OH

## Abstract

**Background:**

Heart and lung transplant patients can develop conditions necessitating general surgery procedures. Their postoperative morbidity and mortality remain poorly characterized and limited to case series from select centers.

**Methods:**

The National Inpatient Sample (1998–2015) was used to identify 6433 heart and 3015 lung transplant patient admissions for general surgery procedures. For a comparator group, we identified 23,764,164 nontransplant patient admissions for the same procedures. Patient morbidity and mortality after general surgery were compared between transplant patients and nontransplant patients. Data were analyzed with frequency tables, *χ*^2^ analysis, and a mixed-effects multivariate regression.

**Results:**

Overall mortality was higher and length of stay longer in the transplant group compared to the nontransplant group. Analysis revealed that hospital size and comorbidities were predictors of mortality for patients undergoing certain general surgery procedures. Transplant status alone did not predict mortality.

**Conclusion:**

Our findings demonstrate that heart and lung transplant patients, compared to nontransplant patients, have more complications and a higher length of stay after certain general surgery procedures.

## 1 BACKGROUND

Heart and lung transplantation has become the standard of care for many eligible patients with end-stage cardiac and pulmonary disease [1,2]. Transplantation rates are now mainly limited by the availability of suitable donor organs. Long-term outcomes have improved as centers have accumulated expertise in managing immediate postoperative complications of transplantation as well as the long-term sequela of chronic rejection with the improved immunosuppression [1].

Heart and lung transplant recipients will often need extra surgical procedures. The morbidity and mortality of common general surgery procedures such as appendectomy, cholecystectomy, hernia repair, and colon and small bowel resection in this patient population are not well known. Lung and heart transplant patients may travel to select centers for transplantation but might require general surgery in their more immediate hospitals. As studies have been limited to case series from select centers [3,4], we sought to better characterize the outcomes of heart and lung transplant patients who undergo general surgery by examining a national database.

## 2 METHODS

### 2.1 Data Source

The Nationwide Inpatient Sample (NIS) database is currently the largest database of all-payer inpatient data [5]. The database is a stratified sample of approximately 20% of US hospitals and contains information on more than 8 million hospital stays per year. This large sample size represents roughly 95% of all hospital discharges and enables analysis of specific patient populations [5].

NIS obtains inpatient data from hospital discharge abstracts and billing records and is able to provide patient demographics, hospital length of stay (LOS), morbidity, in-hospital mortality, and inpatient diagnosis and procedure codes using the *International Classification of Diseases, Ninth Revision, Clinical Modification* (*ICD-9-CM*). The database also provides sampling weights that allow calculation of national estimates. Approval for the use of the NIS data in this study was obtained from the Healthcare Cost and Utilization Project (HCUP) [5].

### 2.2 Study Population

**Fig 1:**
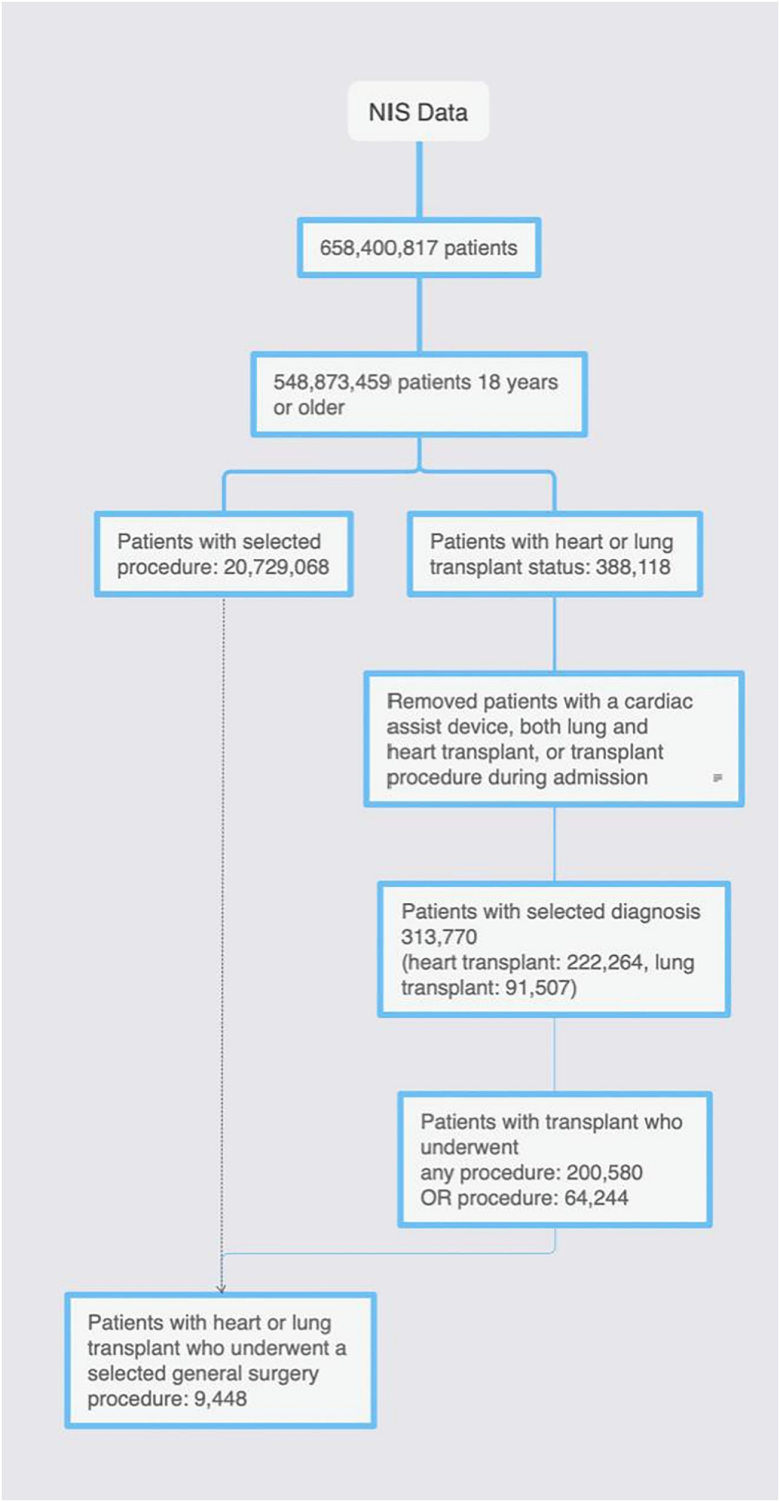
CONSORT flow diagram for patient selection.

Using the NIS database from 1998 through 2015 Q3 (NIS transitioned from *ICD-9* to *ICD-10* in Q3), all inpatient hospital encounters for patients undergoing a general surgery procedure, regardless of transplant status, were extracted [5]. These encounters were then stratified by transplant status into “transplant” (heart or lung transplant) and “nontransplant” cohorts. Admissions under the age of 18 years, with incomplete data, those who had a cardiac assist device, both a heart and lung transplant, or who underwent a transplant procedure during the admission were excluded from analysis. Additionally, if during analysis a sampling stratum contained only 1 sampling unit, it was excluded. A CONSORT diagram demonstrates how the study population was derived (Fig 1).

Diagnosis and procedural codes were selected using the *ICD-9-CM*. Transplant status was identified using diagnosis codes V42.1 (heart transplant) and V42.6 (lung transplant). Procedures considered “general surgery procedures” included appendectomies, cholecystectomies, inguinal hernia repairs, small bowel resections, and large bowel resections. For a comparator group, patient admissions for the same procedures in a nontransplant group (excludes all solid organ transplant including heart, lung, kidney, liver, small bowel) were also identified. Both open and laparoscopic procedures were included except for inguinal hernia repairs, which included only open procedures. Appendix A lists all *ICD-9-CM* codes used.

### 2.3 Outcome Variables

Patient demographics extracted included age, sex, ethnicity, household income quartile, insurance status, and discharge disposition. Baseline comorbidities were identified using the Elixhauser classification system [6]. Primary outcome measures included the postsurgical mortality, postsurgical morbidity, and hospital length of stay for nontransplant, heart transplant, and lung transplant patients undergoing the same procedures. We performed pairwise comparisons between the nontransplant cohort and either the heart or lung transplant cohort. *Postsurgical mortality* was defined as a patient who died after surgery but before being discharged from the hospital because the NIS database does not include any readmission or postdischarge information. Postsurgical morbidity was defined using select using codes from the *ICD-9-CM* and categorized as either a cardiac, pulmonary, operative, or infectious complication. Secondary outcomes were length of time in days between admission and surgery and whether a *delay*, defined as time greater than median time from admission to surgery, was associated with increased morbidity or mortality.

### 2.4 Statistical Analysis

Analyses were performed with raw numbers and weighted by sampling hospitals and strata to reflect national averages [7]. Binary and categorical variables were compared using the likelihood-ratio test statistic for proportions, which was corrected for survey design using the second-order Rao and Scott correction [8]. Continuous variables were compared using 2-sample *t* tests with unequal variance. A multivariate logistic regression with multiple comparisons correction was used to derive factors and their relative weight on postsurgical mortality. Independent variables used for risk adjustment included demographics (age, sex, and ethnicity), hospital size, and Elixhauser comorbidities.

Categorical variables are reported as weighted counts and percentages, whereas continuous variables are reported as ranges or means/medians. Per NIS guidelines, values that contain counts of 10 or fewer were omitted [5]. A *P* value of < .05 was considered statistically significant. All reported *P* values are 2-sided.

Statistical analysis of categorical variables was performed using *χ*^2^ tests, and cohort comparison was performed using *t* tests and analysis of variance. All data analyses were performed using STATA v.14 (College Station, TX) [9].

## 3 RESULTS

### 3.1 Demographics and Frequency of Procedures

A total of 313,770 hospital encounters with existing transplants were identified spanning from 1998 to 2015, 222,264 admissions with an existing heart transplant and 91,507 with an existing lung transplant. From the original sample, 9448 admissions were identified having undergone a general surgery procedure, 6433 heart transplant admissions, and 3015 lung transplant admissions. We also identified 23,764,164 patient admissions for the same procedures in a nontransplant group for comparison.

Heart and lung transplant admission demographics differed significantly from the majority of nontransplant admissions demographics (Table 1). Transplant patients undergoing general surgery procedures were often older (mean age of 57 years transplant vs 51 years nontransplant) and of male sex (68% transplant vs 40% nontransplant). They were also primarily white (81% vs 70% nontransplant), and a majority were Medicare recipients (58% vs 31% nontransplant).

**Table 1:** Demographics by heart and lung transplant status undergoing procedures to those without⁎

Nontransplant			Heart Transplant			Lung Transplant		
*N* = 23,764,164			*N* = 6397		*P*	*N* = 2990		*P*
**Age, y**					< .001			< .001
18–44	8,274,199	35%	731	11%		649	22%	
45–64	7,698,038	32%	3127	49%		1568	52%	
65–84	6,715,845	28%	2515	39%		773	26%	
85 + **Sex**	1,047,441	4%	20	0%	< .001	0	0%	< .001
Male	9,032,674	38%	4844	76%		1505	50%	
Female	14,654,189	62%	1538	24%		1485	50%	
**Race**								< .001
White	13,670,534	58%	4309	67%		2121	71%	
African American	2,100,073	9%	492	8%		137	5%	
Hispanic	2,360,564	10%	297	5%		102	3%	
Other **Primary payer**	1,104,103	5%	180	3%	< .001	50	2%	.002
Medicare	7,962,637	34%	3919	61%		1569	52%	
Medicaid	2,462,756	10%	348	5%		199	7%	
Private insurance	10,888,340	46%	1948	30%		1159	39%	
Self-pay	1,471,160	6%	29	0%		19	1%	
No charge	148,321	1%	15	0%		0	0%	
Other **Income quartile for ZIP code**	770,432	3%	139	2%	.195	42	1%	.042
First quartile	4,623,494	19%	1105	17%		484	16%	
Second quartile	6,011,823	25%	1625	25%		674	23%	
Third quartile	6,028,947	25%	1595	25%		804	27%	
Fourth quartile	6,569,344	28%	1965	31%		988	33%	
**Hospital region**					.503			.378
Northeast	4,587,013	19%	1285	20%		559	19%	
Midwest	5,156,496	22%	1611	25%		851	28%	
South	9,056,758	38%	2150	34%		1002	34%	
West **Elective admission**	4,963,897	21%	1351	21%	.098	577	19%	.311
Nonelective surgery	14,090,102	59%	3627	57%		1725	58%	
Elective surgery	9,000,795	38%	2589	40%		1206	40%	

⁎ Numbers less than 10 were excluded.

**Fig 2:**
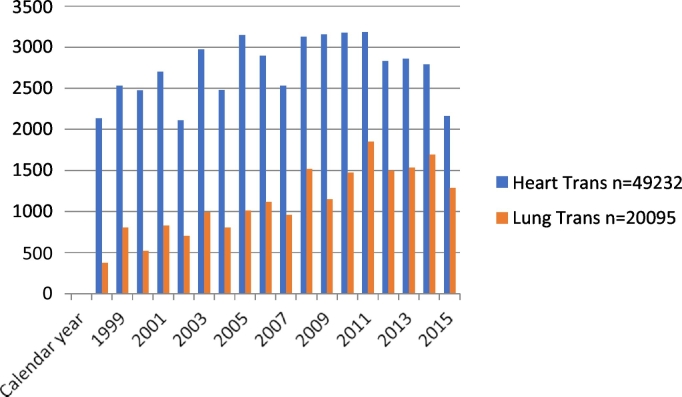
Number of heart and lung transplant patients undergoing elective procedures per year (1998–2015).

General surgery procedures remained relatively constant for heart transplant patients from 1998 to 2015 but steadily increased for lung transplant patients (Fig 2). The majority of both heart and lung transplant patients underwent general surgery procedures as nonelective cases (57% and 58%, respectively). The most common general surgery procedures in heart and lung transplant patients were cholecystectomy (38%, 38%), colorectal resection (19%, 25%), and lysis of peritoneal adhesions (11%, 14%) (Table 2). While admitted in the hospital, heart and lung transplant patients undergoing general surgery procedures had more than 1 procedure compared to the nontransplant group.

**Table 2:** Specific general surgery procedures performed and overall outcomes⁎

	Nontransplant		Heart Transplant			Lung Transplant		
	*N* = 23,764,164		*N* = 6397		*P*	*N* = 2990		*P*
**Number of procedures**					< .001			< .001
1	6,833,681	29%	1332	21%		620	21%	
2	5,766,441	24%	1444	23%		644	22%	
3–5	6,605,777	28%	1780	28%		882	29%	
5–10	3,997,868	17%	1558	24%		731	24%	
10 or more	560,396	2%	284	4%		112	4%	
**Procedure type**								
Inguinal/femoral hernia repair	717,873	3%	603	9%	< .001	68	2%	.290
Cholecystectomy	7,342,580	31%	2415	38%	< .001	1122	38%	.002
Appendectomy	4,586,879	19%	536	8%	< .001	325	11%	< .001
Small bowel resection	1,221,542	5%	430	7%	.015	181	6%	.305
Colorectal resection	4,506,494	19%	1207	19%	.924	755	25%	< .001
Laparoscopy (GI only)	479,705	2%	187	3%	.028	70	2%	.562
Exploratory laparotomy	929,538	4%	291	5%	.307	59	2%	.011
Lysis of peritoneal adhesions	3,979,553	17%	730	11%	< .001	410	14%	.058
**Length of stay, d**								< .001
7 or more	6,909,269	29%	2637	41%		1282	43%	
30 or more **Median length of stay**	494,967 4 days	2%	366 6 days	6%	< .001	141 6 days	5%	< .001
**Disposition of patient**					< .001			< .001
Routine	19,033,478	80%	4622	72%		2106	70%	
Transfer to short-term hospital	161,573	1%	113	2%		68	2%	
Transfer to different facility	1,895,615	8%	516	8%		183	6%	
Home health care	2,082,234	9%	879	14%		496	17%	
Against medical advice	34,078	0%	15	0%		0	0%	
Expired	544,811	2%	248	4%	< .001	137	5%	< .001

⁎ Numbers less than 10 were excluded.

### 3.2 Outcomes of Specific Procedures

Although most patients were discharged home after surgery, transplant patients had lower rates than nontransplant patients (Table 2). Mortality rates were higher in the heart and lung transplant patients compared to the nontransplant group (4% vs 2%, *P* < .001 and 5% vs 2%, *P* < .001 mortality, respectively). Overall complication rates for all general surgeries had greater variability. Heart transplants had the lower rate of total complications, whereas lung transplants had the highest (9% heart transplant, 13% lung transplant, 11% nontransplant). Disparities also existed between the groups for length of stay. Transplant patients were more likely to have longer median LOS in the hospital (6 days for both heart and lung transplant patients) versus nontransplant patients (4 days, *P* < .0001, Table 2). There were also more heart and lung transplant patients who had prolonged length of stay of 30 days or more than nontransplant patients.

Examining specific surgical procedures, small bowel resections had the highest complication rates overall (> 45%) for both transplant and nontransplant patients (Table 3). Transplant patients who had either a cholecystectomy or appendectomy also had higher complication rates. Pulmonary and cardiac specific complications were significantly higher in heart and lung transplant patients versus nontransplant patients in those patients undergoing cholecystectomy, appendectomy, and colorectal resection. Heart transplant patients who had an appendectomy had a 3% operative mortality compared to 1% for nontransplant patients, *P* = .002. Transplant and nontransplant patients had a similar complication profile after hernia repair, with heart transplant patients having fewer cardiac complications than nontransplant patients (6% vs 15%, *P* = .003). Compared to nontransplant and heart transplant patients, lung transplant patients had significantly higher complications (cardiac, pulmonary, and infectious) as well as prolonged length of stay after a lysis of adhesions procedure (Table 3).

**Table 3:** Outcomes for specific general surgery procedures

	Nontransplant		Heart Transplant		*P*	Lung Transplant		*P*
**Inguinal/femoral hernia repair**								
**Any complication**	180,598	25%	114	19%	.094	20	29%	.734
Cardiac complications	110,402	15%	34	6%	.003	11	16%	.976
Pulmonary complications	50,752	7%	38	6%	.711	⁎		
Operative complications	13,206	2%	10	2%	.836	0	0%	.634
Infectious complications	46,391	6%	52	9%	.300	⁎		
Expired Median LOS	13,522 3 d	2%	⁎ 3 d		.731	⁎ 3 d		.302
								

**Cholecystectomy**								
**Any complication**	1,473,291	20%	651	27%	< .001	290	26%	.039
Cardiac complications	585,719	8%	201	8%	.796	139	12%	.017
Pulmonary complications	502,252	7%	297	12%	< .001	108	10%	.118
Operative complications	170,695	2%	95	4%	.015	28	3%	.815
Infectious complications	571,722	8%	240	10%	.062	79	7%	.763
Expired	87,173	1%	42	2%	.220	10	1%	.749
Median LOS	3 d		4 d		< .001	5 d		< .001

**Appendectomy**								
**Any complication**	552,752	12%	146	27%	< .001	102	31%	< .001
Cardiac complications	154,931	3%	33	6%	.098	20	6%	.205
Pulmonary complications	208,668	5%	75	14%	< .001	48	15%	< .001
Operative complications	70,100	2%	⁎			15	5%	.040
Infectious complications	236,122	5%	69	13%	< .001	33	10%	.061
Expired	23,327	1%	14	3%	.002	0	0%	.578
Median LOS	2 d		5 d		< .001	5 d		< .001

**Small bowel resection**								
**Any complication**	568,532	47%	197	46%	.891	84	46%	.974
Cardiac complications	96,070	8%	42	10%	.137	35	19%	.612
Pulmonary complications	228,671	19%	82	19%	.938	30	17%	.748
Intraoperative complications	113,350	9%	32	7%	.540	15	8%	.814
Infectious complications	282,281	23%	97	23%	.916	30	17%	.357
Expired	103,935	9%	53	12%	.212	25	14%	.260
Median LOS	9 d		10 d		.867	10 d		.953

**Colorectal resection**								
**Any complication**	1,446,934	32%	419	35%	.360	325	43%	.001
Cardiac complications	601,635	13%	125	10%	.168	157	21%	.007
Pulmonary complications	552,335	12%	211	17%	.012	118	16%	.204
Intraoperative complications	190,983	4%	58	5%	.694	30	4%	.840
Infectious complications	591,078	13%	182	15%	.348	144	19%	.032
Expired	182,659	4%	76	6%	.061	67	9%	.001
Median LOS	7 d		9 d		< .001	9 d		.015

**Laparoscopy (GI only)**								
**Any complication**	163,104	34%	95	51%	.013	38	54%	.105
Cardiac complications	55,978	12%	10	5%	.218	10	14%	.814
Pulmonary complications	70,958	15%	48	26%	.076	14	20%	.533
Intraoperative complications	25,192	5%	10	5%	.964	⁎		
Infectious complications	67,591	14%	53	28%	.007	24	35%	.033
Expired	69,014	14%	34	18%	.556	15	21%	.444
Median LOS	6 d		9 d		.001	10 d		.386

**Exploratory laparotomy**								
**Any complication**	144,930	16%	52	18%	.659	20	34%	.059
Cardiac complications	42,781	5%	18	6%	.531	10	17%	.027
Pulmonary complications	49,897	5%	⁎			10	17%	.085
Intraoperative complications	37,116	4%	0	0%	.206	0	0%	.507
Infectious complications	46,734	5%	33	11%	.027	⁎		
Expired	9232	1%	⁎			0	0%	.745
Median LOS	2 d		4 d		.001	6 d		.112

**Lysis of peritoneal adhesions**								
**Any complication**	815,344	20%	196	27%	.057	147	36%	.001
Cardiac complications	234,172	6%	72	10%	.042	50	12%	.022
Pulmonary complications	296,978	7%	75	10%	.202	63	15%	.004
Intraoperative complications	223,987	6%	28	4%	.312	15	4%	.417
Infectious complications	285,379	7%	72	10%	.185	57	14%	.014
Expired	58,171	1%	14	2%	.680	14	3%	.127
Median LOS	3 d		7 d		< .001	6 d		< .001

⁎ Numbers less than 10 were excluded.

### 3.3 Correlation Between Time From Admission to Operative Procedure and Predictors of Mortality

We determined the median time from admission to surgery for 4 select procedures for nontransplant patients, heart, and lung transplant patients (median time nontransplant, heart transplant, lung transplant), respectively: cholecystectomy (1, 1, 1 day), appendectomy (0, 0, 0 day), small bowel resection (1, 1, 1 day), and colorectal resection (0, 1, 0 day). *Delay in surgery* was defined as a surgery that occurred after the median time to surgery for that specific procedure. As shown in Table 4, mortality was only increased in patients undergoing small bowel resection whose surgery was delayed greater than the median time determined. Multivariable logistic regression using comorbidities, age, hospital size, and transplant status revealed that hospital size and comorbidities were predictors of mortality for patients undergoing cholecystectomy, appendectomy, small bowel resection, and colorectal resection. Transplant status was not found to be a predictor (Table 5).

**Table 4:** Correlation of delay in surgery with outcomes

	Total Cases	Mortality	Mortality %	*P*
**Cholecystectomy**				
No transplant	2,227,760	23,026	1.03%	
Heart	821	19	2.31%	.097
Lung	375	0	0.00%	.443
**Appendectomy**				
No transplant	588,623	1931	0.33%	-
Heart	79	0	0.00%	.830
Lung	44	0	0.00%	.865
**Small bowel resection**				
No transplant	281,088	27,708	9.86%	
Heart	103	34	33.01%	**.000**
Lung	41	16	39.02%	**.008**
**Colorectal resection** No transplant	1,359,171	97,439	7.17%	
Heart	370	43	11.62%	.258
Lung	297	35	11.78%	.957

**Table 5:** Multivariable predictors of mortality

Predictors	Odds Ratio	95% CI
Transplant Status	0.98	0.69–1.39
Age	1.05	1.05–1.05
Race	1.00	0.99–1.01
Female sex	0.85	0.84–0.87
Hospital bed status	1.12	1.09–1.15
Congestive heart failure	2.09	2.05–2.14
Chronic lung condition	1.27	1.24–1.30
Coagulopathy	4.06	3.95–4.17
Diabetes	0.91	0.86–0.95
Hypertension	0.42	0.41–0.43
Liver disease	2.56	2.49–2.63
Electrolyte abnormalities	2.92	2.85–2.98
Obese	0.55	0.53–0.57
Peripheral vascular disease	2.17	2.11–2.23
Pulmonary circulation disorders	1.53	1.46–1.60
Renal failure	1.77	1.70–1.84
Weight loss	1.74	1.69–1.79

## 4 DISCUSSION

Improved immunosuppressive medications and continued experience in transplantation have resulted in heart and lung transplant recipients living longer. With improved survival, heart and lung transplant patients are at higher risk of being inflicted with conditions that require general surgery [10,11]. Although the index general surgery operation may not necessarily differ between immunocompetent and immunocompromised patients, the ability of transplant patients to recover from complications is limited. Smith et al showed that, in lung transplant patients requiring abdominal surgery, time to surgery of longer than 6 days appeared to be associated with mortality [12]. Our goal was to find guidance on whether these patients should be transferred to tertiary care facilities for their general surgical care or if their acute issues could be dealt locally, avoiding unnecessary travel and delay in treatment. Our study is the first to use a population-based analysis to determine outcomes after such procedures. Our findings are more generalizable than those of previous case series that only report findings from select centers.

### 4.1 Major Findings

We found that heart and lung transplant patients constitute an older subgroup of general surgical patients. These patients also undergo more than 1 procedure while admitted and often have a prolonged length of stay, consistent with transplant patients having more comorbidities [13]. Our study shows that some procedures such an inguinal/femoral hernia repair can by performed safely in either population. For small bowel resections, the outcomes are similar in transplant and nontransplant populations unless there is a delay to surgery. Prior studies have demonstrated that conditions warranting small bowel resection are complex and carry excess morbidity whether or not immunosuppression and transplantation are involved [14]. Results from our study show that for other procedures such as appendectomies, cholecystectomies and abdominal exploration, there is considerable morbidity in heart and lung transplant patients.

Higher morbidity and greater number of operations required for similar diagnosis suggest the higher complexity of similar abdominal problems in thoracic transplant patients. This may not necessarily be a function of their prevalent level of immunosuppression but more likely is a function of cumulative effect of immunosuppression over the lifetime of the transplant. Managing the same disease process in a transplant patient may require one to lean toward being more conservative and choose early operative intervention as opposed to percutaneous temporization interventions. Similarly, intraoperatively, in our practice, we have noticed that surgeons take a more conservative approach in transplant patients, such as use of nonabsorbable sutures for fascial closure and greater use of alimentary tract diversion rather than primarily reestablishing continuity. Prior experience in managing this patient group could be invaluable when facing such a presentation.

### 4.2 Limitations and Strengths

We recognize that there are several limitations to our retrospective data set analysis. Our study is limited to only short-term inpatient outcomes, and postdischarge outcomes such as readmission are not known. The study encompasses 17 years, in which practices may have changed. Because of the small sample size, it was not possible to examine the impact of minimally invasive surgical techniques or volume outcome relationships. Additional factors, such as the specialty training and board certification of the operating surgeon, are not known within the NIS data set, which may be an important determinant of quality of care. We also found that transplant status was not an independent predictor of outcome. We feel that transplant status is a surrogate for comorbidities which are more prevalent in transplant patients. Hence, this may be a construct of our statistical analysis and be a predictor in other models where as many comorbidities are not included. Another limitation is that we are unable to identify when the transplantation occurred, therefore rendering it more difficult when factoring in comorbidities and immunosuppression. Finally, because NIS data are deidentified, hospital admissions rather than individual patients were analyzed. It is therefore possible that a transplant patient may have had more than 1 hospital admission. Given the size of the transplant population (388,118 encounters), NIS's sampling strategies, and variability in patient demographics, this is expected to be a rare occurrence and is adjusted for by not reporting on groups with fewer than 10 individuals.

Despite these limitations, our study is the first ever to report a population-based analysis of general surgical outcomes in heart and lung transplant patients with a meaningful sample size. We conclude that all general surgical procedures should not be taken lightly in this population given the increased potential for morbidities and prolonged length of stay.

### 4.3 Recommendation

The postoperative morbidities and prolonged length of stay after some of these procedures argue that these transplant patients are better served at tertiary care centers [[15], [16], [17]]. Our analysis hinted at this, as hospital bed size was a predictor of mortality. The ability to rescue from complications is an increasing well-known factor that determines mortality. Larger institutions are likely better equipped to deal with postoperative complications as well as manage immunosuppression regimes. Specialized medical and surgical units dealing with heart and lung transplant patients can facilitate postoperative care as well as ensure that posttransplant protocols are being followed. Transfer to these specialized facilities, in our opinion, should be considered in patients, particularly if patients are stable. Patients requiring emergent operative intervention, such as that for small bowel obstruction, should not, however, be denied surgery because the patients comorbidities’ and not transplant status determine outcome.

## Figures and Tables

**Table:** 

Study Population	*ICD-9-CM* Code
Heart transplant status	V42.1
Lung transplant status	V42.6
**Procedure type**	
Inguinal/femoral hernia repair	53.00–53.05, 53.10–53.17, 53.21, 53.29, 53.31, 53.39
Cholecystectomy	51.21–51.24
Appendectomy	47.0, 47.01, 47.09, 47.1, 47.11, 47.19
Small bowel resection	45.61, 45.62, 45.63
Colorectal resection	17.31–17.39, 45.71–45.79, 45.8–45.83, 48.40–48.49, 48.5–48.59, 48.61–48.69
Laparoscopy (GI only)	54.21
Exploratory laparotomy	54.11
Lysis of peritoneal adhesions	54.51, 54.59
**Morbidity**	
***Cardiovascular***	
Supraventicular arrhythmia	427.30, 427.31, 427.32, 427.0
Myocardial infarction	410.0–410.9, 411.81, 413.0–413.9, 997.1
Postoperative stroke	997.02
Deep venous thrombosis	451.1, 451.2, 451.81, 451.9, 453.2, 453.8, 453.9, 453.40–453.42
Pulmonary embolism	415.1, 415.11, 415.12, 415.19
***Pulmonary***	
Pneumonia	481–486, 482.0–482.3, 482.41, 482.49, 482.80, 482.83, 482.9, 486, 997.3, 997.31, 997.32
Postoperative acute respiratory insufficiency	518.5
Postoperative acute pneumothorax	512.1
Postoperative pulmonary edema	518.4
Pulmonary collapse	518.0
Empyema with and without	510.0, 510.9
Fistula	
Mechanical ventilation	96.70, 96.71, 96.72
Noninvasive ventilation	93.90
***Infectious***	
Sepsis/shock	995.91, 038, 995.92, 999.3, 998.0
Urinary tract infection	599.0, 590.9
Postoperative wound infection	998.51, 998.59
***Intraoperative complication***	
Accidental puncture or laceration, complicating surgery	998.2
Bleeding complicating procedure	998.11
